# Prospects of cold plasma in enhancing food phenolics: analyzing nutritional potential and process optimization through RSM and AI techniques

**DOI:** 10.3389/fnut.2024.1504958

**Published:** 2025-01-15

**Authors:** M. Anjaly Shanker, Sandeep Singh Rana

**Affiliations:** Department of BioSciences, School of Bio Science and Technology (SBST), Vellore Institute of Technology, Vellore, India

**Keywords:** total phenols, bioactive potential, cold plasma, artificial neural networks, efficacy

## Abstract

Consumption of plant-based food is steadily increasing and follows an augmented trend owing to their nutritive, functional, and energy potential. Different bioactive fractions, such as phenols, flavanols, and so on, contribute highly to the nutritive profile of food and are known to have a sensitivity toward higher temperatures. This limits the applicability of traditional thermal treatments for plant products, paving the way for the advancement of innovative and non-thermal techniques such as pulsed electric field, microwave, ultrasound, cold plasma, and high-pressure processing. Among these techniques, cold plasma would be an operative choice in plant-based applications due to their higher efficacy, greenness, chemical exclusivity, and quality retention. The efficiency of the plasma process in ensuring the bioactive potential depends on several factors, such as feeding gas, input voltage, exposure time, pressure, and current flow. This review explains in detail the optimization of process parameters of the cold plasma technique, ensuring greater extractability or retention of total phenols and antioxidant potential. Response surface methodology (RSM) is one of the common techniques involved in the optimization of these course factors. It also covers the convention of artificial intelligence-based methods, such as artificial neural networks (ANN) and genetic algorithms (GA), in evaluating the data on process parameters. The review critically examines the strengths of each optimization tool in determining the optimal process parameters for maximizing phenol retention and antioxidant activity. The ascendancy of these techniques was mentioned in the studies regarding fruit, vegetables, and their products, and they can also be applied to other food products.

## 1 Introduction

Food is a well-recognized source of nutrients and bioactive components essential for sustaining life and promoting health. In recent years, food has emerged as a functional component with additional physiological benefits, including the prevention or delay of illnesses and health conditions. A new diet-health concept with a major emphasis on positive aspects of food beyond basic nutrition is followed by different groups of people irrespective of age, culture, and social domains (1). Current research is focused on exploring the health-promoting, disease-preventing, and protective capabilities of major food groups. Amid these stands, plant-based diets comprising fruits, vegetables, herbs, seeds, and so on have immense health potential with basic nutritional value and incidence of other bioactive components. The bioactive potential of these food groups has sparked interest in their health-promoting roles, encouraging their incorporation into regular diets. The presence of bioactive compounds in food is considered essential, as they can influence the body holistically or target specific tissues, contributing to overall health and wellness. These compounds do not fit into the category of nutrients as they are not considered elemental, but their existence offers a constructive effect on the body in terms of their biological activity. The major classes of bioactive components in plant foods are phenols, flavonoids, carotenoids, plant sterols, tannins, and other sulfur-based compounds (2). They are present in multiple forms, such as esterified, glycosylated, hydroxylated, and so on, in plant-derived food items and have an influential role in overall systematic functioning (3). Fruits and vegetables are known for their functional and antioxidant bioactive profile, which contribute to antitumor, anti-cardiac, anti-inflammatory, anti-mutagenic, anti-cancer, and neuroprotective potential (4).

Polyphenols are one of the important classes of secondary metabolites, and they have a large range of structural and functional possibilities in fruits, vegetables, and other plants. Known as natural antioxidants, they are secondary metabolites of plants structured with an aromatic ring consisting of 2 or more hydroxyl moieties. They are water-soluble fractions having a molecular weight of around 500 to 4,000 Da and have more than 8,000 types of recognized orientations (5). They range from simple phenolics, such as hydrobenzoic acids, to large high-molecular polymers such as tannins. These compounds are considered to be important determinants of agricultural produce, as they are physiological, sensorial, morphological, and nutritional.

The phenol content of produce is dependent on environmental factors such as sun exposure and soil type and varies according to genetics, maturity, post-harvest operations, and other factors. Choosing the right methods for technological processing is crucial to maintaining the availability of high-quality agricultural produce throughout the supply chain (6).

### 1.1 Phenols & their classes

**Figure 1:**
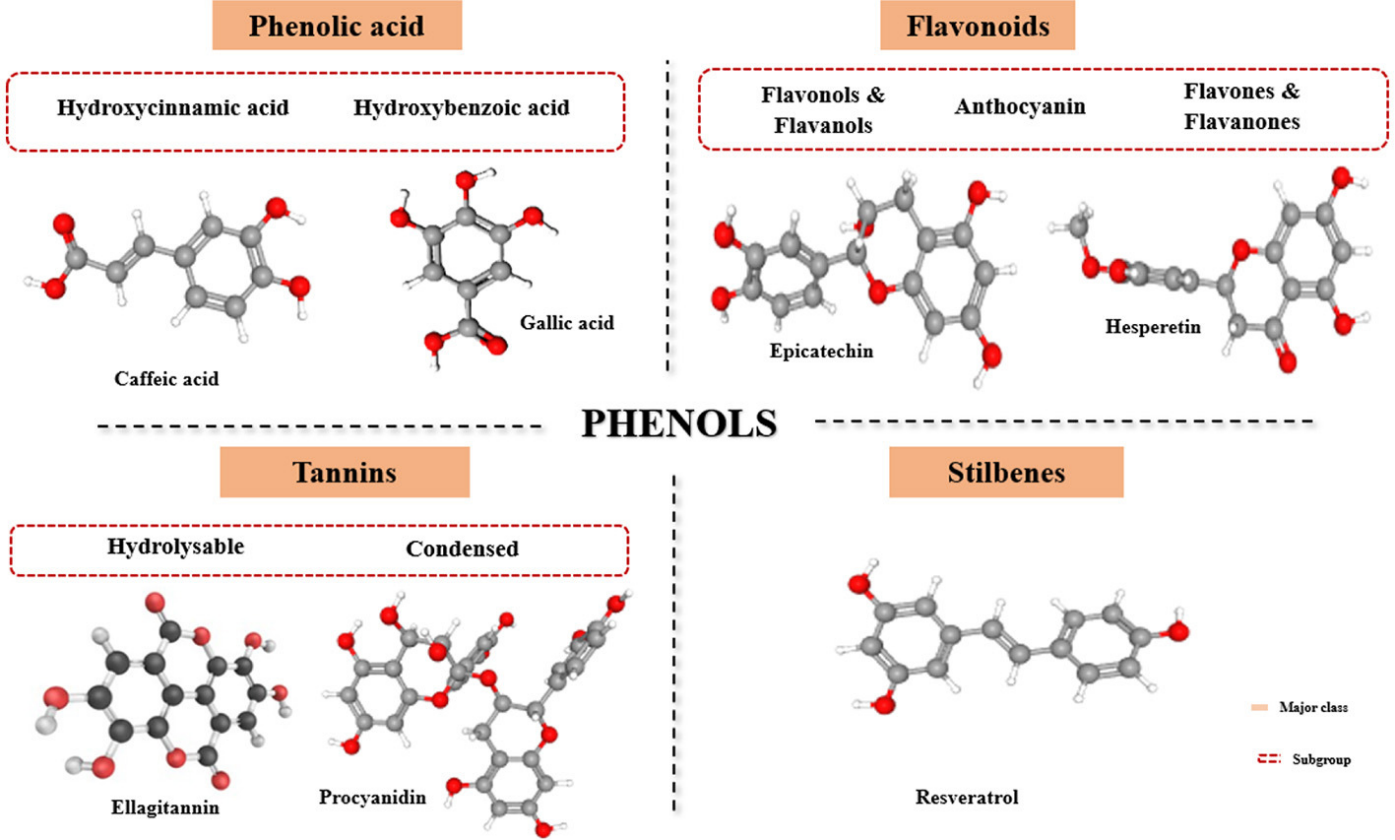
Summary of major phenols in agricultural produce.

Phenolic compounds include not only an array of molecules with a basic polyphenol structure but also those with a single phenol ring-like phenolic acids. They are divided into different categories, conferring the number of phenol rings present and the structural elements that bind these rings to each other (Figure 1). The major classes of polyphenols are flavonoids, phenolic acids, lignans, tannins, and stilbenes (4). Flavonoids, the most abundant type of phenolic compounds, feature a basic C6-C3-C6 structure that includes 15 carbon atoms. The characteristic construction of flavonoids comprises two aromatic rings (A & B) combined by a 3-carbon bridge in the form of a heterocyclic ring (C) (7). Varying substitution arrangements of ring C result in the cataloging of flavonoids as flavones, flavonols, flavanols, flavanones, flavanonols, isoflavones, and anthocyanidins. Similarly, substitutions such as oxygenation, glycosylation, acylation, alkylation, and sulfonation to the aromatic rings (A & B) create variations within each category of flavonoids. Owing to their high redox potential, flavonoids are important antioxidants present widely in agricultural produce, and they have a positive role in reducing diseases such as cancer and heart-related issues (8). Among the different classes of flavonoids, the structurally diverse and commonly found class of compounds are flavonols and flavones. The structural configuration of these classes is a basic flavonoid structure with a double bond between the C2-C3 position and an oxygen atom at the C4 position with an addition of a hydroxyl group at the C3 position for flavonols. Flavanones are characterized by a saturated three-carbon chain and a C4 position substituted by an oxygen atom, whereas isoflavones constitute a diphenyl propane structure with the B ring positioned at C3 (9). Although flavanones are predominantly found in high concentrations in citrus fruit, they are also present in tomatoes, grapefruits, licorice, berries, and aromatic plants such as mint (10).

The basic structure of anthocyanins is anthocyanidins, which consist of aromatic ring A bonded to oxygen-substituted heterocyclic ring C, which in turn is linked with ring B by a carbon-carbon bond (11). They are water-soluble pigments characterized in agriculture and produced by red, purple, and blue color profiles. They are rich in red grapes, raspberries, cherries, strawberries, and berries. Six common anthocyanidins present in plants are petunidin, cyanidin, delphinidin, pelargonidin, peonidin, and malvidin (12). Phenolic acids represent one-third of dietary phenols and are categorized into two major subgroups: hydroxybenzoic and hydroxycinnamic acids. The former group, which has a characteristic C6-C1 structure, is commonly represented by gallic acid, vanillic, p-hydroxybenzoic acid, and so on. In contrast, the latter group with caffeic, ferulic, sinapic, and coumaric acids as cinnamic acid derivatives are represented by three carbon side chains (C6-C3) (13). While there is a very low distribution of hydroxybenzoic groups in plants, excluding certain red fruits and onions, the incidence of hydroxycinnamic acids is widespread in fruits and vegetables, with blueberry, cherry, plum, and kiwi having the highest content of this class (3).

Tannins are high molecular compounds that are further grouped as hydrolyzable and condensed tannins. The most widely recognized forms of condensed tannins are epicatechin and catechin, which are polymerized flavonoids in nature (14). Hydrolysable tannins are gallic acid derivatives that can be easily degraded by biological systems. Tannic acid is an example of this class, comprising eight to 10 molecules of gallic acid. Tannins have described competence of substantial structural variations and are considered potential metal chelators, biological antioxidants, and protein precipitating agents (15). Lignans are widely distributed compounds in the plant domain derived from oxidative dimerization of two phenylpropane units. Based on the positioned oxygen in their structural skeleton, oxidation levels of side chains, and cyclization pattern, the compounds are classified into eight different subgroups, including furan, furofuran, dibenzyl butane, and so on. Lariciresinol, pinoresinol, matairesinol, and secoisolariciresinol are commonly found in lignans in plants and are distributed in fruits, vegetables, seeds, and in beverages such as juices, coffee, and wine (16). Stilbenes appear in plants in cis and trans configurations with major dietary sources such as grapes, wine, juices, and peanuts. Resveratrol, one of the common forms of stilbenes, is known for its chemopreventive, anti-inflammatory, anti-proliferative, and antioxidant properties and includes grapes, berries, pines, and so on as their major sources (17).

### 1.2 Importance of the cold plasma technique

Thermal treatments are the primary traditional methods used in the post-harvest treatment of vegetables and fruits to extend the shelf life of the produce. Traditional techniques can reduce the nutritional and functional potential of produce by altering its component profile, which is a significant drawback. Research on innovative non-thermal technologies gained popularity and interest in this context, contemplating the demand for good, safe, functional, and nutritionally superior food products in the consumer markets (18). Cold plasma is a non-thermal technique that ensures the quality of food products with maximum retention of quality and safety. The reliability and versatility of this green technique have promoted the application of the plasma technique and have been reported to have a substantiated potential in food packaging modification, decontamination, toxin removal, enzyme inactivation, and even wastewater treatment (19). The efficiency of the process in ensuring the bioactive and functional profile of fruits and vegetables is dependent on several factors such as feeding gas, input voltage, exposure time, pressure, current flow, and so on. Optimization of process parameters and application of different optimization techniques ensure maximum quality and nutritive retention, along with a detailed understanding of the effect of each parameter on the efficiency of the process. This review aims to explore the possibilities of plasma technique in retaining the phenolic profile of vegetables and fruits along with the optimization of process parameters with different statistical methods such as response surface methodology and artificial intelligence-based techniques such as artificial neural networks (ANN) and genetic algorithms (GA).

Mentioned as the fourth state of matter, plasma denotes quasi-neutral ionized gas comprising ions, reactive species, photons, and free electrons in addition to their excited or fundamental states with a net neutral charge in the system (20). Similar to the phase change from solid to liquid to gas, increasing the energy input beyond a certain threshold in the gas phase leads to particle excitation, resulting in the formation of plasma. The general classification of a plasma system based on thermal equilibrium includes thermal and low-temperature (non-equilibrium) plasma (21). The former condition involves plasma generation by heating gases to higher temperatures, thereby attaining a thermal equilibrium within the species. The latter is further classified into quasi-equilibrium, where the constituents are in local thermal equilibrium (100°C−150°C), and non-equilibrium plasma, where they exist in thermal non-equilibrium (<60°C). The low-temperature, non-thermal, or non-equilibrium plasma with a lower temperature range is termed cold plasma (22). Any source of energy that can ionize gases can be employed in the generation of these increased energy levels of plasma in a system. It can be generated with the help of thermal, electrical, optical, radioactive, or electromagnetic energy sources applied through a gas system, resulting in the atoms' dissociation, excitation, and ionization. However, the widely accepted and applied generation mode involves electric and electromagnetic sources. In addition to the source and type, another important point of consideration in plasma generation is the selection of operational gas. Common gases involved in the generation process are oxygen, nitrogen, and carbon dioxide, and there is a possibility of encompassing noble gases such as argon and helium into the system as operational gases (23). The selection of gas involved in plasma generation, in turn, affects the efficiency and economic feasibility of the process, making it one of the important parameters of the process.

The different approaches actively involved in plasma generation are dielectric barrier discharge (DBD), corona discharge, microwave (MW), radio frequency plasma (RFP), atmospheric plasma jet (APPJ), glow discharge (GDP), gliding arc discharge (GAD), and resistive barrier discharge (RBD) plasma (24). Among these, DBD and plasma jet are the common techniques involved in the treatment of food particulates. However, this commonality is not always applicable, as the selection of a system of operation should be dependent on the product characteristics. A dielectric plasma system involves the generation of plasma by applying a voltage between two known electrodes maintained at a small distance from each other. This economically feasible and viable option can be employed at a range of pressures, voltages, and frequencies under different gas sources (25). The versatility in application parameters of the DBD system presents it as a common generation type with a wide application range. The plasma jet system setup comprises a nozzle with two concentric electrodes through which the carrier gas passes and is subjected to a high voltage of 100–250V. Common carrier gases used in the system are oxygen, carbon dioxide, helium, or a mixture of gases treated at higher frequencies. Microwave discharge plasma system setup encompasses a power source, standing wave radiometer, circulator, quartz tube, and microwave-to-plasma applicator (26). Here, the generation of plasma and excitation of gas electrons is aided by the origination of microwaves at a general frequency of 2.45 GHz. This plasma system functioned at a pressure range of 1–10^5^ Pa and does not require an electrode to generate plasma. The application of different systems, in turn, results in the effective excitation and ionization of gases, leading to the interactions between reactive species and food components. These interactions act as the sole reason for the modification of the functional, chemical, nutritional, and microbiological profile of the food.

## 2 Mechanism of action on phenols

The relevance of cold plasma in food, including its interactions and its effect on food components, remains an exploratory field of study. A proper interpretation of the interactions of food and plasma components has a decisive role in integrating the system in the treatment of any food. In the case of phenolic compounds, this mechanism of action is related to the synergistic effect of generated plasma reactive species on the structural conformities of phenolic compounds in food (23). There are different explanatory approaches related to the variations in the phenolic profile owing to the cold plasma treatment. Oxidation caused by reactive oxygen species is a primary mechanism affecting phenol concentrations (27). Interaction results in the alteration of structural features of phenol fractions, resulting in the formation of new carbonyl and carboxylic groups. Alteration in chemical structure could be due to intense surface oxidation, which affects the antioxidative and functional properties of the fraction. Hydroxylation reactions that cause structural modification in benzene rings owing to the reactive species result in a gush of related interreactions, such as the formation of carbonyl radicals and generation of phenoxyl radicals altering the phenolic content and functional activity of the samples (28). For instance, superoxide radicals formed by the dissociation of oxygen molecules in the plasma environment exhibit oxidative degradation as well as double-bond structural disruption in different phenolic compounds owing to the formation of carboxylic and carbonyl compounds (29). Concurrently, the reactive plasma species also includes a reactive nascent oxygen species created by the dissociation of molecular oxygen by electron effect. This powerful oxidizing atomic form reacts with the benzene ring of phenol, forming primary diol products such as catechol and resorcinol, which, in turn, react with atomic oxygen, forming secondary products (30).

The presence of ozone is inevitable due to the high reactivity of the compounds, which leads to the hydroxylation of the benzene ring, forming hydroquinone. These dihydric phenols then undergo ring cleavage, producing major intermediates such as oxalic acids and glyoxalic acid, and are known to cause modifications in the cell wall that enhance phenolic compounds (31). Furthermore, hydroxyl radicals, which are reactive fragments generated by plasma, initiate the hydroxylation of the benzene ring to form hydroquinone (32). This is followed by an oxidation reaction that leads to the production of intermediate benzoquinone and, subsequently, the formation of fumaric, maleic, and oxalic acids as end products. Consequently, reactive species from cold plasma can affect cell viability by causing the rupture or disruption of the cell membrane, thereby affecting the availability of phenolics. Another significant approach resulting in phenol concentration variations is the nitration process, where the accumulation of nitrates or nitrites leads to the formation of nitrophenols, affecting the functional profile and polymerization reactions, forming larger polymeric structures of phenols modifying solubility and bioavailability of these compounds (31). Approaches on the effect of plasma on phenylalanine ammonia-lyase, an important enzyme in synthesizing phenol concentration, influence the phenol activity in samples. Inactivation or modification of enzymes encompassed in the synthesis pathway of phenols can directly alter the phenolic content of a product.

The effect of plasma on phenol and bioactive components exhibits a varying trend involving positive and negative inclinations. Compared to thermally treated and untreated samples, there is a rise in phenol concentration of plasma-treated samples with dependency on various parameters, including time, gas flow rate, and so on, which is explained in detail in the coming sections. There is a reported increase in phenolics by 64%−69% owing to the plasma treatment at 4–5s in cloudy apple juice (33). Parallel to these results, a more than 10% increase was induced by atmospheric plasma treatment in cashew apple juice (34). There are reported affirmations on the positive inclination of plasma in apple juice (35), acerola juice (36), sour cherry juice (37), and pomegranate juice (38).

Contrary to these samples, a decreasing trend in phenolic activity was noticed in the case of prebiotic orange juice (39), white grape juice (40), and strawberry fruit (41). These variations are highly dependent on the product and the process characteristics, and it is important to identify the significance of each parameter to maximize the process efficacy. The outcome of plasma on phenolic concentrations in various fruits and vegetables is given in Table 1.

**Table 1:** Details on the effect of plasma on phenol concentrations.

**Sample**	**Treatment conditions**	**Observations**	**Advantages**	**References**
Tomato juice	DBD plasma Voltage: 60 kV Frequency: 50 Hz Time: 10, 15 min	Rise in phenol content by 4% at 10-min treatment. A slight decreasing effect was reported in the case of 15-min treatment	•Higher retention of nutrients •Color improvement •Minimal impact on physicochemical properties	(42)
Apple juice	Jet plasma Frequency: 25 kHz Time: 30–120s	The increase in time was favorable, with treatment time at 120s resulting in a 14% increase in phenolic content	•Microbial resistance •Non-destructive in nature and shorter treatment times •Retention of sensory and nutritional properties	(35)
Banana slices	DBD plasma system Voltage: 4.8–6.9 kV Frequency: 12–22 kHz Time: 35–155 s	Total phenol & flavonoid content exhibited an increasing trend, mounting the treatment time and voltage. The optimum treatment condition was at 6.9 kV for 46s	•Enhanced bioactives and antioxidant activity •Retention of vitamin content •Effective in enzyme inactivation	(43)
Strawberry fruit	DBD plasma system Voltage: 60 kV Frequency: 50 Hz Time: 10–30 min	Treatment at 15 min was found to have a positive effect on the phenolic content	•Quality preservation •Enhanced bioactive potential •Synergistic effects	(41)
Cherry	DBD plasma system Voltage: 40, 60, 80 kV Time: 60, 80, 100, 140 s	Higher voltages have a detrimental effect on the overall phenolic content, with 60 kV treatment having no significant effect. Treatment time does not negatively affect the phenol content	•Enhanced shelf life •Retention of key quality attributes such as color, firmness •Enhanced bioactive potential	(44)
Fresh-cut pitaya fruit	DBD plasma Voltage: 60 kV Time: 5 min	The cumulative trend in the phenolic content was observed at the prescribed parameter range	•Promoted levels of antioxidants •Better product quality	(45)
Tomato pomace	DBD plasma Voltage: 120 V Frequency: 60 kHz Time: 15 min Working gas: air, argon, helium, and nitrogen	Higher phenolic content was observed in the nitrogen and helium plasma-treated samples compared to the control, whereas air and argon did not exhibit much difference.	•Enhanced phenolic content and antioxidant activity •Synergistic mechanisms •Retention of physicochemical quality	(46)
Orange juice	Jet plasma Frequency: 25 kHz Time: 30–120s	More than 9% increase in phenolic concentration owing to treatment time of 120s	•Microbial stability •Minimal processing and reduced treatment times •Effective retention of nutritional and sensorial parameters	(35)
Chokeberry juice	Jet plasma Power: 4 W Frequency: 25 kHz Time: 3, 5 min	No substantial change was noted in the treatment	•Polyphenol stability •Reduction in aerobic bacteria and yeast counts	(47)
Fresh cut apples	DBD plasma Frequency: 12.7 kHz Time: 15–120 min	No difference in phenolic content after 30 min of treatment. A decrease in phenolic content by 9%−33% was observed after the 30- and 120-min treatment period.	•Reduced enzyme activity and browning reactions •Increased shelf life	(48)
Apple juice	DBD plasma Power: 30–50 W Time: 0–40 s	Higher time periods and power had a detrimental effect on phenolic content value. Lower voltage values at 30 and 40 W did not have a substantial effect	•Faster treatment •Faster inactivation of microbial populations •Color retention •Enhanced phenol concentration	(49)
Blueberry	DBD plasma system Voltage: 12 kV Frequency: 5 kHz Time: 0–90 s	Increase in phenol content with an increase in treatment time	•Faster treatment •Reduction in decay rate •Retention of firmness value	(50)
Siriguela juice	Glow discharge plasma Time: 5–15 min Nitrogen flow rate: 10–30 mL/min	A gas flow rate of 30 mL/min showed a reduction in phenolic content by 30%. A 58% increase in phenolic content was exhibited at a 15 min and 20 mL/min gas flow rate.	•Color stability •Enhanced microbial stability •Enhanced bioactive components	(51)
Orange juice	DBD plasma system Voltage: 230 V Frequency: 50 Hz Time: 15–60 s	Phenolic content was affected only after 60s of treatment. Till 45s of treatment, the total phenolic content of the samples was retained	•Retention of physicochemical and sensorial factors of the sample •Smaller treatment times •Enhanced oligosaccharide content	(39)
Pomegranate juice	Plasma jet Frequency: 25 kHz Time: 3, 5, 7 min Gas flow rate: 0.75, 1, 1.25 dm^3^min^−1^	A moderate treatment time of 5 min resulted in an increase of phenolic content by 33%. There was a small decrease observed with augmented flow rate	•Fast, accurate, and non-invasive treatment •Better stability of the sample	(38)
Carrot juice	DBD plasma system Voltage: 60–80 kV Frequency: 50 Hz Time: 3, 4 min	No significant change between the different evaluation parameters. Maximum retention of phenolics was found with 70 kV treatment for 4 min	•A superior and viable alternative to thermal treatment •Maximum inactivation of enzymes and microbes •Enhanced stability of the juice	(52)
Okra pods	DBD plasma system Power: 750 W Frequency: 20 kHz Time: 5–30s	With an increase in time, there is a noted difference in total phenol content of 5%, 13%, and 20%.	•Increase in chlorophyll beta content •Faster treatment •Better flavonoid retention	(53)
Tomato juice	DBD plasma system Voltage: 220 V Frequency: 10 kHz Time: 0–5 min	Slight reduction in total phenol content by an increase in time of more than 15%	•Faster treatment times •Effective in fungicide degradation •Better quality product	(54)
Carrot juice	DBD plasma system Voltage: 8, 10, 12 kV Frequency: 18 kHz Time: 0–5 min	Total phenolics of the sample did not show much difference at 8 kV treatment, followed by a decrease at 10 kV and a further increase in total phenolic content	•Superior to traditional heat treatment •Extended shelf life •Better quality retention	(55)
Mango	Gliding arc plasma Power: 600 W Gas flow rate: 2 to 8 L/min Time: 5, 10 min	An increase in treatment time had a negative impact on total phenolics. The increase in gas flow rate resulted in an initial phenolic content increase of 5 L/min, followed by a reduction in parameters.	•Reduction in pesticide residues •Increase in carotenoid content	(56)
Avocado pulp	DBD plasma system Time: 10–30 min Gas flow rate: 10 to 30 mL/min	An increase in treatment time and lower gas flow rates positively influence the phenolic content of the sample. Treatment at 10 mL/min for 30 min was reported to have an increase of 18% in the phenol content of the sample.	•Increased carotenoid levels •Retention of quality attributes	(57)
Persian lime fruit juice	Gliding arc plasma Power: 300 W Flow rate: 10 mL Frequency: 50 Hz Time: 30–120s	Rising trend in the total phenolic content till the 60s of treatment, followed by fluctuations with an increase in time. The higher phenolic content was observed in the treatment at 60s.	•Enhanced post-harvest storage life •Retention of juice yield and other sensorial attributes •Better microbial stability	(58)
Yam slices	Glow discharge plasma Power: 500 W Time: 90–180s	CP treatment showed retention in the phenol content of the sample with the highest phenolic content at the 90s of treatment.	•Reduction in peroxidase activity •Enhanced antioxidant activity	(59)
Tomato juice	DBD plasma system Voltage: 40, 45 V Time: 3, 4, 5 min	Significant reductions in the phenolic content of the samples were observed with a rise in voltage and treatment time. Higher values were reported at 40 V for 3 min of the treatments, followed by 45 V for 3 min.	•Reduction in fungicide levels •Prolonged shelf life •Microbial stability	(60)

## 3 Decisive parameters in cold plasma treatment

Laterally, with the generation systems, the levels of different parameters, such as input power, gas flow rates, treatment periods, and sample placement, influence the positive and negative variations observed in the food system (61). Power or voltage applied across the electrode has an unswerving influence on the reactive species generated. The dielectric breakdown of air is known to be achieved by the application of electric field power at 30 kV/cm (31). The power or voltage should be maintained in a plasma system so that it is sufficient for the generation of plasma, but it should not have a detrimental effect on the quality properties of food. The effective parameter range depends on the product characteristics, and it is known to have positive inclinations at a lower range of voltage applied. An increase and retention in the phenol concentration was reported in the case of cherry samples treated by cold plasma at a voltage of 40–60 kV (44). Elevated voltage levels resulted in a detrimental effect on the total phenol concentration of the sample, which emphasizes the viability of moderate treatment conditions. A similar trend was observed in the case of apple juice, which underwent jet plasma treatment, conveying an elevated power-dependent decrease in phenol concentrations (35). Polyphenol content increased from 30.57 to 43 mg/100 g of fresh weight in banana slices with an increasing voltage from 4.8 to 6.9 kV. The antioxidant activity and total flavonoid content also followed a similar increasing trend with a change in voltage (43). Plasma species and their reactivity play a vital role in the interactions that happen post-treatment in food products. Gas flow rates directly impact the reactivity of these generated plasma species, and a reported increase is shown in this with an increase in the discussed parameter. As in the case of power applied, moderate conditions of gas flow rates are favorable, with higher or elevated rates tending to shorten the residence time of active species and thereby reduce the system efficacy. On the contrary, very low flow rates may not be adequate for initiating the interaction of plasma species with a short half-life. In this case, higher flow rates will increase the likelihood of interactions in the plasma environment (5). Retention of phenolics was safeguarded in acerola juice samples when the flow rate was maintained at 10 mL/min, and a further increase in flow rate (up to 20 mL/min) induced a decreasing trend in phenolic concentration (36). Siriguela juice showed a varied result of positive correlation with a gas flow rate of up to 20mL/min followed by a reduction in the phenol concentration of 30% with a surge in the flow rate to 30 mL/min (51). Ten mL/min was considered to be ideal in plasma treatment of avocado pulp samples, with an increase in phenolic content by 5%−18%. Lower gas flow rate values were found to safeguard a milder effect on the degradation reactions, upholding the level of phenolic content in the sample. Higher flow rates in the range of 20–30 mL/min were not favorable in the retention of phenolics in the sample, and there was a substantial decrease reported when the gas flow rate was increased to 30 mL/min (57). Observations exhibited highlight the fact that an ultimate elucidation on the feasible parameter range is not conceivable, as the inclinations vary according to the product and process characteristics.

Another important parameter under consideration is plasma generation frequency, as it is directly associated with the excitation behavior of ions from the plasma system. A maximum phenolic activity of 720 mg gallic acid equivalent/g was reported in apple cubes treated with a dielectric plasma system at a frequency of 60 0Hz (62). A higher excitation frequency (900 Hz) was reported to have a detrimental effect on the phenolic profile of the treated sample. Differing from the stated results, plasma-treated samples showed higher phenolic content at lower and higher (200 and 960 Hz) frequency ranges. An increase in frequency levels of plasma treatment has substantially affected the phenol content and antioxidant activity of camu-camu juices (63). The decrease in phenol concentration was observed at moderate frequency levels of 420–628 Hz in treated camu-camu juice. A similar trend is observed in the case of the antioxidant activity of the samples, as the phenolic content has a contributory effect on the mentioned parameter. Variations in results can be viewed regarding the difference in the synergistic profile of parameters such as power and frequency or time and frequency of the plasma system and the generated plasma species. Even the characteristics of the treated sample will act as a combinatorial factor in the efficacy of the plasma system. Feed gas is a significant parameter that alters the nature of the plasma reactive species and the food interactions employed in the system. For example, the change in oxygen concentration significantly affected phenolic content during the CP jet treatment of blueberry juice (64). The stable nature of argon gas does not affect the chemical composition of the food product, retaining the phenolic content and antioxidant capacity. A rise in O_2_ concentration had directed a positive change in phenolics with maximum concentration at a higher percentage of O_2_ in the system. The combination of inert gas and oxygen resulted in decreased oxidative degradation of functional compounds, maintaining their concentration and antioxidant properties. Higher phenolic content was observed in the nitrogen and helium plasma-treated samples compared to the control, while air plasma treatment did not differ much (46). Treatment time is one common and conducive parameter that ensures the effectiveness of any treatment in food particulates. In the case of plasma treatment, the time designed for treatment will endorse the extent of the interactive nature of the reactive plasma species. Reactive plasma species should have sufficient time to interact with food components, positively influencing the functional nature or engrossing in degradative inter-reactions that closely affect the food characteristics at elevated times. The prolonged processing time of CP treatment has displayed higher phenolic content in blueberry juice (60), and this was in accordance with studies on mandarin peel (65) and cashew apple juice (35) with maximum retention of phenolic content.

Meanwhile, reported results showed a decrease in phenolic content from 2.52 to 1.93 g/L in orange juice, owing to plasma exposure for more than 60s (39). Higher treatment time also showed a negative impact on the phenolic content of white grape juice (40), acerola juice (36), and sour cherry marasca juice (37). These contradictory findings emphasize the importance of a detailed understanding of the product and active species interaction to effectively comprehend the plasma process. Along with the product-reactive species interactions, it is important to understand how the plasma-treated samples behave during the shelf-life period. The phenolic content and antioxidant activity of the plasma-treated samples followed an increasing trend during the initial storage period, followed by a decreasing inclination compared to the control sample. However, the superiority of the plasma-treated samples in terms of phenolic content was sustained in whole blueberry samples for a period of 40 days (50). Similar tendencies over the storage periods were reported in the case of potato tubers treated with a plasma jet reactor for 0–40s time. The total phenolics of the samples decreased with longer storage hours after displaying an increasing trend during the initial days. After 8 days of storage, comparative analysis showed that the phenolic content of the potato slices treated for 20 were 18% higher than the control samples (66). The treatment at 20 and 30 maintained a phenol content value superior to the control samples throughout 16 days of storage, whereas treatment at 40s was found to be inferior. Time dependence on the efficiency of the process has already been discussed, and there is a visible influence of the parameter variation even in keeping the quality of the product. More investigations are necessary to understand the parameter fluctuation range affecting the process and the product.

In addition, external factors such as relative humidity, sample matrix, and pH have an effect on the properties of reactive species and the interactions happening in the system (67). When considering the case of sample properties, starting from the state of the food product, there will be changes in the interaction. Plasma species interactions with a solid matrix will differ entirely from a liquid matrix, with higher area or contact points. Within the solid matrix, there will be differences according to the surface microstructure & porosity, component profile, and even the moisture content (68). This difference exists in regard to the relative humidity and pH of the treatment environment, as the type and effectiveness of the reactive species are, in turn, added to the variations in these parameters. Supplementary to the discussed aspects, the source type, material of construction, and packaging variations, i.e., whether the process involves an open treatment or in-package treatment of plasma, influence the effectiveness of the interactions, thereby influencing the process variables.

## 4 Process optimization & modeling

Statistical methods can evaluate the extent of influence of intrinsic parameters involved in the process dependency representations are performed with the help of models. This study proposes the right experimental model by following different screening levels, aligning, and optimizing data values.

### 4.1 Response surface methodology

RSM is a set of statistical and mathematical models ascertained on the fit of the polynomial model to data, which should exhibit the conduct of the whole data set for making statistical predictions. This approach effectively evaluates, optimizes, conceives, and refines the processes where a response(s) is controlled by the variation of several other variables (69). In the evaluation process, the value of the response is envisaged based on the factors considered, and the intricate interplay between the presence of several independent variables is comprehended. The following result encompasses a mathematical model having an ideal combination of factors to deliver optimized results. Selecting an appropriate experimental design is crucial for RSM analysis, as the design of the experiment determines the points at which the response should be assessed. The most used experimental design models in the food industry are central composite design (CCD), central composite design (CCRD), Box-Behnken design (BBD), multilevel factorial design, and so on. The application of RSM techniques in food research follows a longer trail with applications around food drying, extraction, encapsulation, enzymatic hydrolysis, blanching, and certain food formulations (70).

#### 4.1.1 Central composite design

CCD is an effective and most commonly applied form of design that provides equitable evidence for investigating the lack of fit of data without including larger experimental runs. Applied design has the competency to predict both linear and quadratic models with high quality, and they can also be employed to study factors at three or four levels. In the case of cold plasma treatment, CCD is believed to be the most common design form employed in optimizing the parameters related to the quality of the final product (71). A central composite design with 16 experimental trials, three independent variables including gas flow, sample volume, treatment time, and one output parameter was used to evaluate the efficacy of gas phase plasma treatment in sour cherry Marasca juice (35). Based on the analysis, the major factorial change was induced by variation in the treatment time of the plasma technique, while the other two factors remain minor contributory. Optimal treatment conditions for maximum phenolic retention were 3 min, 3 mL, and 0.75 L/min of treatment time, sample volume, and gas flow. Ideal values of plasma parameters in retaining the phenolic content of ginger samples were determined by the interaction between dependent and independent variables in CCD design (72). The independent factors involved in the study are power, time, pressure, and rotation, and each of them is assigned three coded levels with a total of 37 trials in the system. A linear effect of each parameter was visible in the retention of TPC in the plasma-treated samples. With increased treatment power and time, there is a negative inclination in TPC values, ranging from 35 mg to 14 GAE/g. The rotation of samples was found to be effective in retaining the phenolic content, having an observable difference from the non-rotated sample. The factors involved in the study have distinct and combined effects on the final phenol content of the sample. The optimized treatment conditions after 1 min at a power level of 50 W and pressure of 0.65 mbar retained higher concentrations of phenols in the sample.

Central composite face-centered design (FCCD) is one of the different designs within CCD that evaluate and optimize the parameters in cold plasma treatment of blended beverages (73). FCCD with two independent parameters, including treatment time and voltage, are involved in the evaluation process. The optimized treatment conditions recognized through RSM analysis reflected a delicate steadiness between the analyzed parameters. Both voltage and time had an influential role in the antioxidant activity of the sample, exhibiting a plummeting trend with an increase in the combined parameter values. Plasma treatment at a voltage of 18 kV for 1.75 min was optimized to balance the antioxidant profile and microbial safety. FCCD design with six center points, four cube points, and four axial points was used to assess the effect of DBD cold plasma on the phenol content and antioxidant activity of green tea leaves (74).

Three levels of treatment time and power were taken as independent variables to understand the effect these variables have on the final output. The influence of power and time on the TPC values of the samples was significant, but the independent effect of time on the phenolic profile was found to be marginal. A predominant effect on the phenolic profile emanates from the variation of power in the treatment and its combination with treatment time. The optimized treatment parameters were obtained as a power of 15 W for a treatment time of 15 min for achieving an increase in phenol content by 41% and antioxidant activity by 41.04%. Analogous to plasma treatments, CCD is also employed in assessing the effect of different independent parameters such as time, temperature, and solvent-to-solid ratio on three responses during the extraction of phenols from potato peels (75). Experimental design with 20 runs, eight factorial points, and six axial and central points each provided an optimum parameter value of 30 min of treatment time at a temperature of 75°C and 60 mLg^−1^ solvent-to-solid ratio.

#### 4.1.2 Central composite rotary design

Central composite rotary design is considered an alternative to the factorial design and can quantify the relationship between controllable inputs and resultant responses. The key advantage of the process design over other models is its capacity to optimize multiple operational variables with a small experimental data set. These possibilities assisted in the application of this design format when the values of certain investigations were altered by experimental error (76). CCRD has been found to be efficient in predicting and formulating the interactions of input parameters with the possible responses of cold plasma treatment (77). CCRD, with five levels and two independent factors, including time and voltage, is studied against six other parameters, including phenolic content and antioxidant activity. Linear and square regression coefficients of two independent variables are found to impact the particular responses with optimized parameters at a range of 35 kV voltage for a treatment time of 510 seconds. The phenolic content value of the treated sample showed an initial increase up to a certain point, followed by a decreasing trend due to the rise in voltage and time. The linear coefficients of both voltage and time are positively correlated with phenolic content, whereas there is a negative correlation of square coefficients of parameters. In the CCRD design of the experimental evaluation of cold plasma parameters in kiwifruit juice, the juice depth was utilized as an additional independent parameter (78). A set of 20 experiential runs with six output parameters was involved in the experiential pattern, and there was a linear dependency of TPC over-voltage and time. Voltage and depth show a positive dependency on the final phenolic content, and negative dependency was observed on treatment time. There is a positive correlation between the phenolic content of the sample and the voltage and time values up to a certain point. Beyond this point, the phenolic content decreases. In operations such as cold plasma, where each independent parameter can insinuate a certain degree of variation in totality, it is always preferred to choose these methods or designs where multivariate analysis is feasible on a larger scale.

#### 4.1.3 Box-Behnken design

BBD was first designed by Box and Behnken with the ability to go with fewer experimental runs and has a definite set of factorial combinations from the 3 k factorial design. This three-level design has an economic ascendancy over other models, as the employed experimental runs can be a minimum (76). The Box–Behnken experimental design effectively predicted the independent parameters' possible interactive effects during the cold plasma treatment of kiwi turbid juice (79). Here, voltage and treatment time have an added authority over the phenolic responses of the product more than the third factor. The phenol content of the samples reported a decreasing trend with increasing treatment voltage and time. The optimum experimental conditions were reported to be at a treatment voltage of 12 kV and a volume of 18 ml for a treatment period of 1 min. Parallel to these findings, the optimization efficacy of the BBD design model in ultrasound-assisted extraction from cherry fruit was reported. With 29 experimental runs, four independent parameters, and two dependent parameters, the experimental data were placed in a second-order polynomial equation to understand the fit (80). Independent variables showed a significant quadratic effect in the phenolic content of the plasma-treated samples, whereas the effect of linear variables was insignificant. The optimal conditions with maximized response variables were found to be a 26 mLg^−1^ solvent-solid ratio, a solvent concentration of 58%, and 28% amplitude for a treatment time of 31 min. Even though economic feasibility is associated with the process, it comes under the category of the least explored design aspect regarding cold plasma treatment and needs more experimental data to conclude the degree of efficacy.

### 4.2 Model fitting

The application of different model systems is common in the scientific research field, and it is also invariable in the case of the food research segment. The feasibility and effectiveness of each model in a system are deeply correlated with the parameters and processes involved (Table 2). In the case of cold plasma treatment of agricultural produce, regression models are commonly involved in checking the feasibility of predicted responses. The validation and selection of the model are based on their fit and alignment with the experimentally obtained results. The model's fit is validated by ensuring a significant *p*-value of <0.05, no significant lack of fit, a high R^2^ value, and a difference of <0.2 between the predicted and experimental values (69). This predictive evaluation involves the mathematical representation of response surfaces as regression equations. Depending on the correlation between the response surface and the input variables, the equations can be first or second order in description (71). Response surface plots of 3 min cold plasma treatment of sour cherry juice followed a 2^nd^ order polynomial equation, and there are linear, quadratic, and interaction coefficients predicting the desired final response value (37). All four variables exhibited a quadratic effect on the phenol concentration of the sour cherry juice and were reported to have significant interaction between the variables. The experimental values obtained after evaluation were found to be 98% of the predicted phenolic responses, emphasizing the model's success in envisaging the interactive correlations. From the discussed instances, we have gained an understanding that time has a significant impact on the efficiency of cold plasma. Therefore, optimizing the process study with a broader range of time values can enhance the model's efficacy. Similar is the case with the regression equation based on plasma treatment in tender coconut water (81). The parameter range is so limited that the interactive effects of the independent factors are difficult to recognize. The proposed model of this study was effective as there were adequate data points within the proposed smaller range of study. A good fit concerning the experimental and predicted data is denoted by a higher R^2^ value and a *p-*value of lack of fit higher than 0.05. A higher value of R^2^ can be considered the strength of the quadratic representative form of cold plasma treatment of sour cherry juice. RSM analysis of cold plasma treatment data showed an R^2^ value higher than 0.95 and a *p-*value below 0.05, emphasizing the uniformity of predicted and experimental values (80). The model's fitness in defining the data set can be attributed to its characteristic aspect in judging the response structure over a range of input variables. The 2^nd^ order polynomial regression equation expressing the relationship between the input variable and phenolic response is given as:


(1) $$\begin{array}{l} Y=-14.15\;+\;3.13\;x_{1}+0.01x_{2}\;+5.12\times 10^{-6}\;x_{1}x_{2}\\ \;-0.043\;{x_{1}}^{2}-2.3\times 10^{-5}{x_{2}}^{2} \end{array}$$

**Table 2:** Details on RSM analysis of cold plasma treatment variables.

**Sample**	**Experimental variables**	**Optimized conditions**	**Equation**	**Pros & cons of the selected model**	**References**
**Kiwi turbid juice**	Voltage Time Volume	Voltage: 12 kV Time: 1 min Volume: 18 ml	Y_2_ = 2.84 – 0.11A + 0.79B – 0.11C + 0.11AC + 0.19A^2^ + 0.52B^2^	•Easy to interpret & consolidate •Works well with the linear relation of parameters with phenol content •Issues may arise when dealing with complex relations •Sensitive to outliner data	(79)
**Tender coconut water**	Voltage Time	Voltage: 18 kV Time: 2.85 min	Y_1_ = 26.59–0.85x_1_- 0.77x_2_ −0.23 x_1_x_2_ – 0.34 ${\text{x}}_{1}^{2}$ + 0.11 ${\text{x}}_{2}^{2}$	•Simple in form •Have moderate flexibility •Assumes a linear relationship between independent and non-independent variables •Increased risk of overfitting •Need of more resources	(81)
**Ginger**	Power Time Pressure Rotation	Power: 50 W Time: 1 min Pressure: 0.65 mbar Rotation: On	Y_4_ = 8.49–0.4619A −1.04B+ 0.1556 C-0.1318D – 0.5973AB – 0.2191AC+ 0.0714AD- 0.0282BC- 0.0234BD- 0.0203 CD+ 0.4917A2 - 0.0564B2−0.8279 C2	•Provides interaction effect analysis •Reduction in experimental runs •Provides perceptive visualization of connected variables •Does not provide mechanistic insights into the processes affecting the sample properties	(72)
**Green tea leaves**	Power Time	Power: 15 W Time: 15 min	Y1 = 840.87 −15.83A −56.00B + 2.75AB + 2.52B^2^	•Reduction in number of experiments saves time, resources, and cost •Generates equivalences that envisage extraction efficiency and antioxidant activity under different experimental conditions •Sensitive to input data	(74)
**Pineapple juice**	Voltage Time	Time: 510 s Voltage: 35 kV	Y_TPC_ = −14.15 + 3.13 x_1_ +0.01 x_2_ + 5.12 × 10^−6^ x_1_ x_2_ - 0.043${\text{x}}_{1}^{2}$ −2.3 × 10^−5^ x_2_^2^	•Comprehensive optimization •Generated outcomes can aid in scaling up and process optimization functions •This might result in oversimplification of the non-linear effects of DBD plasma treatment •Accuracy depends on the provided data quality	(77)
**Siriguela juice**	Gas flow Time	Gas flow: 20 mL/min Time: 15 min	Z = 820.69–102.76x+ 6.0 x^2^ + 25.23 – 0:55y^2^ – 013xy	•Systematic optimization of cold plasma parameters •Effective interpretation of the plotted data in understanding the underlying interactions •Effect the fit of the data if we consider more complex interactions •Limited to specific parameters involved in the study	(51)
**Kiwifruit juice**	Voltage Juice depth Time	Voltage: 27.6 kV Time: 6.8 min Juice depth: 5.2 mm	Y_i_ = c_0_ + c_1_ x_1_ +c_2_ x_2_ +c_3_ x_3_ + c_4_ x_1_ x_2_ +c_5_ x_2_x_3_ + c_6_ x_3_x_1_ + c_7_ ${\text{x}}_{1}^{2}$ +c_8_ ${\text{x}}_{2}^{2}$ + c_9_ ${\text{x}}_{3}^{2}$	•Exploration of interactive effects on juice quality •Additional validation is required owing to parameter specificity •Limited scalability	(78)
**Pear cactus fruit**	Power Time	Power: 750 W Time: 40 min	Y_i_ = 57.458 – 0.076X_1_ + 1.028X_2_ + 2.171X_1_X_2_ + 1.101${\text{x}}_{1}^{2}$ + 2.171${\text{x}}_{2}^{2}$	•Involves fewer trial runs in predicting the outcomes of the conditions tested effectively •Customization potential •Identifies correlations but limits in apprehending the underlying mechanisms •Limited in managing data complexity	(82)
**Red globe grapes**	Gas flow rate Time Voltage	Gas flow rate: 2.87 SLPM Time: 1 min Voltage: 3.36 kV	Y_i_ = +0.8440 + 0.0006 X_1_ + 0.0094 X_2_ + 0.0075 X_3_ + 0.0010 X_1_ X_2_ −0.0002 X_1_ X_3_ – 0.0077 X_2_ X_3_ −0.0032 ${\text{x}}_{1}^{2}$ – 0.0053 ${\text{x}}_{2}^{2}$ 0.0020 ${\text{x}}_{3}^{2}$	•Effective in predicting responses related to the sample quality •Data complexity is affecting the efficacy of a selected representative model •Sensitive to the input data quality	(83)

These coefficients, x_1_ (voltage) and x_2_ (time), represent the two independent variables, and y is the response, in this case, phenol content (77). Cross-product terms in the equation are responsible for model interactions between the explanatory variables. The single variable accounts for the linear effect, and the squared terms account for the quadratic effects and are responsible for response curvature. The positive values in the regression equation represent a synergistic effect, whereas a negative term denotes an antagonistic effect on the final parameter. Linear and squared regression coefficients of voltage and time display a considerable effect on the response of the samples, with marginal influence from the interaction coefficients. Linear coefficients exhibit a positive correlation in contrast with negatively correlated interaction coefficients. The coefficient of determination of treatment values was around 0.945, explaining the goodness of fit and the parallel nature of the target and predicted values.

Table 2 gives an explanation of the polynomial equation involved in cold plasma treatment of food products. With an R^2^ value of 0.928, the quadratic regression model equation exhibited a good fit between the predicted and experimental values of cold plasma treatment of ginger samples (72). The model was developed with a broader range of parameter values, which has helped in understanding the interactions when it comes to higher ranges of values. However, the iterations, or the number of data points between the ranges, appear to be small and can be included to provide a more profound understanding of the interactions. Cold plasma treatment evaluations have already shown us how the dependency of the process changes with the alterations in process parameter values. Analogous to the results, the quadratic model representing the phenol retention efficacy in cold plasma treatment of green tea leaves (74) has made efforts to have a wider range of independent variables, contributing to the model efficacy. The proposed model is based on 3 data points in the case of both variables, which could have affected the efficiency of the optimization process.

Furthermore, including additional lower data points within a modified broader range would have positively impacted the proposed model. The model representing the DBD plasma modification of kiwifruit juice (78) presented an R^2^ value of 0.95 with quadratic terms of voltage and time, showing a significant effect in the response variable. Representing a larger data point in two variables, the process evaluates several interactive parameter combinations, which positively affect the suitability of the proposed polynomial equation. In the case of the third variable (time), there is a scope for extending the lower range of the data set, thereby improving the proposed model.

### 4.3 Artificial intelligence based techniques

Artificial intelligence has now become prevalent across a different range of industries and sectors, such as healthcare, finance, manufacturing, transportation, and retail markets. It has also speckled down to applications in agriculture and food industries. The conceivable role of AI in the food industry is to leverage developmental opportunities to enhance product quality, safeguard consumer needs, manage supply chains, improve production, and drive a sustainable and successive industrial stature. The basic idea behind the AI technique revolves around the emulation of the human thinking process, learning ability, and storage of knowledge. It involves the development of algorithms and computational models that enable machines to process and analyze large amounts of data, identify patterns and relationships, and make predictions or decisions based on that analysis (84).

The system incorporates a diverse array of algorithms, such as artificial neural networks (ANNs), genetic algorithms (GAs), logic programming, cognitive science, expert systems, swarm intelligence, and fuzzy logic (FL). These algorithms facilitate process estimation based on the sample set and the final output. Notably, FL and ANN are specifically used for predicting and classifying parameters, which contributes to increased yield, reduced production costs, and enhanced safety and quality of the final product (85).

#### 4.3.1 Artificial neural networks

ANN is a powerful and unique modeling system that outshines in capturing non-linear relationships and indicates superior predicting capabilities. The elementary structure of an ANN consists of an input layer, a few hidden layers, and a yield layer. Each structural layer consists of a set of neurons interconnected by a link, and communication between these neurons is achieved by a value assigned to each link called weight. It is one of the artificial intelligence-based elements fabricated to mimic the human brain and evaluate the data through learning and interneuron connections (85). The system's expediency and capability have accelerated feasibility studies on its application in areas such as quality analysis, food safety management, image analysis, classification, prediction of parameters, and modeling of various processing operations.

**Figure 2:**
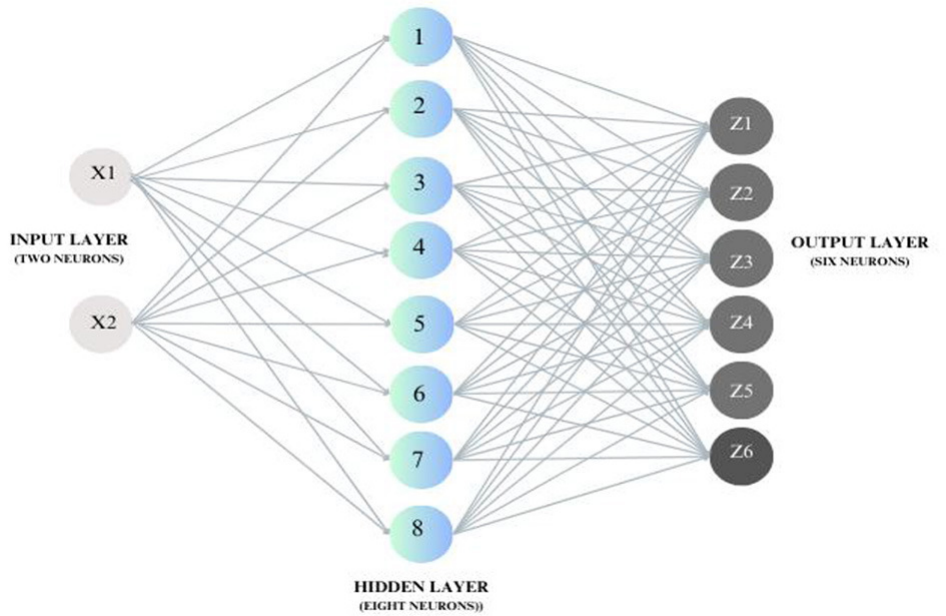
A schematic diagram of the ANN model used with two input layers, one hidden layer, and six output layers.

The ANN model benefitted from the prediction and modeling of the relation between input and output parameters in the non-thermal plasma treatment of pineapple juice (77). A feed-forward neural network with an input layer of two neurons (time and voltage), a single hidden layer, and an output layer of 6 neurons, including total phenol content and total antioxidant activity (Figure 2), was used in the analysis. The number of hidden layers is determined through an iterative trial-and-error method. During this process, the hidden layer of the training with the maximum R-value and minimum MSE value is selected. A total of 12 experiment data sets for the program were categorized as 70% for training, 15% for testing, and 15% for validation with transfer functions for output and hidden layers as linear transfer functions (purelin) and tangent transfer functions (tansig). The objective of data set categorization in the ANN system is to ensure the network's robustness and normalize the network error, which is considered to be a greater positive of the system than others. High correlation coefficient (R) and low mean square value (MSE) were used to confirm the feasibility of the number of hidden layers employed and the model's appropriateness. The final evaluating structure consisted of two neurons in the input layer, eight in the hidden layer, and six in the output. Training, testing, and validation correlation coefficients were found to be 0.9986, 0.9997, and 0.9983, with an R-value of 0.9996 for the overall developed ANN model, ensuring the system's credibility in evaluating and predicting data. An analogous R-value of 0.9997 was obtained for ultrasound-assisted extraction of polyphenols from Meghalayan cherry fruit via an artificial neural network (80). Here, the input layer consisting of four variables, eight neurons in one hidden layer, and the output layer with two neurons constituted a feasible design for forecasting this experimental data with a minimum MSE value of 0.61. Input, hidden, and output layer combinations, the transfer functions involved, data categorization, and the number of neurons in each layer depend on the characteristics of the data that's been handled and the way it is handled (Table 3). Parallel to these results, a categorization of the data set as 70% for training, 15% for validation, and 15% for testing was employed in the 3:10:10 ANN model for thermosonication of sohshang fruit juice. The obtained value of the coefficient of variation was around 0.97, which explains the acceptability of the predicted fit. There is an excellent agreement between the predicted and experiential values in the regression analysis plots, justifying the significant relationship between the data sets (86).

**Table 3:** Summary of the reported transfer functions involved in cold plasma data modeling.

**Structural design**	**Transfer functions involved in the process**
Input layer	•f(p) $=\frac{1}{\;1-e^{-p}}$
Hidden layer	•f(p) = $\frac{1}{1-e^{-p}}$ •(n) = $\frac{2}{1+e^{-2n}}-1$ •(n) = $\frac{2}{\left(1+\exp\left(-2^{*}n\right)\right)-1}$ •(n) = $\frac{1}{1+\exp\left(-n\right)}$
Output layer	•(n) = n •f(p) = $\frac{1}{1-e^{-p}}$

A good correlation of 0.99947 was obtained during ANN analysis of plasma treatment of tender coconut water (81). In this structured evaluation of parameters, it is important to fix the number of hidden layers chosen, as too many layers may cause overfitting of data. In contrast, too few may cause slower processing of data in the network (77). A structured 2-7-2 model was utilized for cold plasma treatment with one of the output neurons as antioxidant activity. This design showcases the significance of interneuron connections between the layers, weight, threshold values, and the matrix arrangement within the system's input, output, and hidden layers. The R^2^ values for individual processes were 0.9993 for training, 0.9998 for validation, and 0.99976 for testing (81). The correlation coefficient value states the positive relationship between the actual and predicted values of the experiential learning procedure; the values showing closeness to 1 always emphasize a positive correlation in the study. A minimum R^2^ value above 0.80 is considered a standard for all optimal models, and with these higher correlation coefficient values, the model's accuracy is ensured. Training, validation, and testing of microwave processing parameters on enzyme activity and phenolic content of tender coconut water also showed similar correlation coefficients of 0.99967, 0.99945, and 0.99987 for the tests, respectively (87). A neural network type of 3-9-1 topology efficiently evaluated the total phenolic content in strawberries. The result validates the effectiveness of threshold transfer functions of purelin in the output layer and transig in the hidden layer in achieving an R^2^ value of 0.9806 and a low MSE value of 0.00470, respectively (88).

In the ANN modeling of CP-independent parameters, an experimental data distribution of 80%, 10%, and 10% was used for training, validation, and testing, respectively, utilizing a multilayer feed-forward neural network design (78). This network consists of an input layer with three neurons (time, voltage, and juice depth), two hidden layers, and an output layer with six neurons, including measures for total phenolic content and total antioxidant activity. The first and second hidden layers employed hyperbolic tangent and log sigmoid transfer functions, respectively. After several trials, the number of neurons in the hidden layers was increased to ten, achieving a maximum R-squared (R^2^) value of 0.999. Correspondingly, the R^2^ values for the training, validation, and testing phases each also reached 0.999.

The regression analysis data exhibited all regression lines closer to 1. The error histogram of the evaluated value showcases that the maximum data, in this case, exhibits a very low error of 0.00142, verifying the prediction performance of the process evaluation. With a low error value of 0.0527, the error histogram was divided between 20 vertical bins between −3.424 and 1.6 in predicting the quality attributes of pineapple juice after non-thermal plasma treatment (77). These error values predict the difference between the predicted and target values. Having a low error value for the maximum data among the analyzed values signifies the correctness of the data involved. Considerable data points amount to an analogous error value of 0.080006, indicating the accuracy in modeling the data related to the thermosonication of sohphie fruit juice (89). Regression analysis of the data also showed a greater correlation between the predicted and the actual values throughout overall data sets. The correlation between the actual and predicted data values always delivers a sense of reliability and accuracy in the system's application in evaluating the experimental array of data. Although the related data on ANN looks assuring and precise, added data evaluation, further experimental data sets, more permutations, and combinations are needed to ensure the process's applicability, dependability, and accuracy.

#### 4.3.2 ANN-GA optimization

A genetic algorithm (GA) recognized as a global search engine in combination with ANN modeling will assist in plummeting the complexity of the problems and discovering optimum process conditions. The evaluation process helps obtain an optimum value with maximum accuracy and minimum error (90). The process commences with choosing a set of initial populations followed by their corresponding set of fitness functions evaluated based on Darwin's theory or principles of genetics involving selection, crossover, mutation, and reproduction. The best among the present chromosomes is selected for the upcoming generation and combined during crossover to create progenies. Different food processing operations, such as extraction, drying, puffing, and roasting, have followed the application of a combination of ANN-GA in evaluating, modeling, and optimizing the available data set. The input parameters of cold plasma treatment in pineapple juice were evaluated by coupling ANN with this built-in optimization tool (77). The fitness function involved in the optimization process is based on your data set and the output parameters considered in the function. The fitness function of CP treatment in pineapple juice is given by Equation 1 with three input parameters and six output parameters, including the total phenol content of the treated juice after treatment.


(2) $$\begin{array}{l} F=Y\left(1\right)+\frac{1}{\;1+Y\left(2\right)}\;+Y\left(3\right)+Y\left(4\right)+Y\left(5\right)+Y\left(6\right) \end{array}$$

The optimization of the ANN model was completed after 68 generations amid voltage, juice depth, and treatment time values of 30 kV, 5 mm, and 6.7 min. The predicted and validated responses of the process remained comparable, with a small error value of 0.83%. This shows the accuracy of the optimized values, and in comparison with the control values, these results exhibited the retention of TPC content of the plasma-treated sample. Ultrasound-assisted extraction of total phenols and other phytocomponents from dragon fruit peel was modeled and optimized by the combined ANN-GA technique (91). The output parameters or responses were selected based on the highest fitness function, and the optimum value parameters of the process were found to be 59.96°C, 60%, 20 min, and 25:1 for ultrasonic temperature, solvent concentration, treatment time, and solvent-to-solid ratio, respectively. The optimization process was validated by conducting experiments at predicted ideal conditions, and these obtained values are equated with the predicted data set to identify the associated deviation. Relative deviations between the predicted and target values of optimized parameters in ultrasound treatment were 1.2–2.05, which emphasizes the accuracy of the optimization process. GA optimization has reported an ideal parameter combination of temperature at 40°C, the amplitude of 50% for a treatment time of 60 min for thermosonication of *Elaeagnus latifolia* fruit juice, and the results were found to closely match with real outcomes based on optimized results (86).

Optimal values for parameters of CP-treated kiwifruit juice were found to be 38 kV and 631 s after 176 generations of the ANN-GA optimization process. The optimal values of the process exhibit good retention of phenolic compounds and reveal comparable phenolic and antioxidant content on evaluation with thermally treated samples. The lower percentage errors (1.00%) between the predicted values and the targeted experimental values show the suitability of the fit in the cold plasma technique. ANN coupled with GA aided in predicting the optimum parameter values for the thermosonication process of sohphie fruit juice after 15 iterations in the study (89). The results obtained with optimized parameter values were in conjunction with the projected denominations produced by the ANN model with the least error values associated. The optimized parameters for the ultrasonic treatment were a 40°C temperature, 50% amplitude, and a treatment period of 60 min.

Precision in the GA optimization process is achieved by either minimizing or maximizing the GA output parameters to secure a high-fitness functional value. For the ANN-GA-optimized process of cold plasma (CP) treatment in tender coconut water, minimal error values were noted between the predicted and experimental values. Optimal results were achieved at a voltage of 18 kV and a treatment time of 2.85 min, with the predicted scavenging activity around 26.71%. Similarly, extraction process parameters of pectin from sunflower heads were determined using a coupled ANN-GA with a second-order polynomial equation serving as the fitness function. The maximum yield of pectin was comparable to the predicted results obtained from GA optimization, affirming the adequacy of these models. The reliability and accuracy of these predictions are evidenced by minimizing error values, underscoring the effectiveness of GA as a robust optimization tool for managing experimental CP data.

## 5 Conclusion and future prospects

Cold plasma has gained significant popularity and interest among stakeholders in recent years due to its innovative and substantial potential in processing and preserving agricultural produce. While most of the studies and findings revolve around the possibility and viability of plasma in rendering a safe product, the likelihood of the technique in functional and quality characteristics of food particulates is still nascent. Phenolic fractions are important bioactive compounds in agricultural produce that can be regarded as contributory aspects or determinants of antioxidant potential. The prospects for quality modifications of plasma in agricultural produce are directly correlated with the generated active plasma species, processing conditions, and induced food interactions. Reaction chemistry is crucial in understanding the intensity of functional and nutritional modification of food. Reported outcomes of oxidative degradation, hydroxylation of benzene rings, nitration of phenols, and so on are proposed as the conceivable explanations prompting the functional and structural modification of the phenolic profile. There is still a prerequisite need to understand and elucidate the intricate molecular-level interactions behind these food interactions to increase the efficiency of the whole process.

Cold plasma treatment, without a doubt, has been reported to have a positive impact and the upper hand in the retention of bioactive components compared with traditional thermal treatments. The degree and inclination of this plasma impact depend on different process parameters such as treatment time, power, voltage, and gas flow rate. The system's mode of action and efficiency can also vary according to the generation mechanism and food characteristics. Careful selection of treatment parameters is essential to achieving desired results and maintaining system efficacy. One aspect of accomplishing this is conducting extensive research encompassing parameter range variations and closely inspecting the effects these variations have on product characteristics. This is where the application of modeling, prediction, and control tools comes into the picture. Comprehension of a feasible set of parameters significant to the study is made possible by the application of statistical-based experiment design in different processes involved. Statistical-based methods such as RSM could effectively help us understand the cross relationships between these regulating parameters and predict the possible permutations and combinations related to the plasma treatment of agricultural produce. The application of AI techniques such as ANN and GA optimization in cold plasma can also widen the prospects of application by disregarding the complexity of the selection of parameters, thereby contributing to the efficacy of functional improvement in agricultural produce. This possibility of statistical method lacks practice in investigating parameters related to the plasma treatment of agricultural produce. The studies related to the application of statistical methods such as ANN and RSM in cold plasma investigations were found to be imperative in concluding the influential parameters and their effects on product functional quality. Further research on plasma treatment using statistical and predictive tools will help in drawing conclusions about its efficiency and enhancing our understanding of the process.
